# Fossil Man

**Published:** 1863-09

**Authors:** 


Fossil Man.—Not long since, in some remarks upon the *Antiquity of Man*, we alluded to the discovery of M. Boucher de Perthes in the ancient drift deposit of the Somme valley, of flint implements wrought by the hand of man and associated with the bones of extinct mammalia, namely, Elephas primigenius, the cave-bear, cave-hyæna, &c. On the 28th of March this same indefatigable gentleman, while engaged in his explorations, was fortunate enough to find the only link wanting to complete the chain of evidence as to the authenticity and importance of his previous labors, for in the high level beds of Moulin-Quignon near Abbeville, and beneath a layer of rough flints, he found a human jaw. This discovery, of so vast importance both to anthropology and to geology, necessarily produced great excitement in the scientific world, and strong doubts as to its authenticity were expressed by men of science in England. On May 15th, a congress of palæontologists was held at Abbeville, and the whole evidence in the case was thoroughly examined. The English men of science present, Dr. Falconer, Mr. Prestwich and Prof. Busk, who had previously been loud and strong in their statements as to its falsity, were obliged to admit the accuracy of the views of such men as Milne-Edwards, Quatrefages, Lartet and others, who had never doubted the authenticity of the specimen. The following are the conclusions arrived at, unanimously, by the commission:—"1. That the jaw found at Moulin-Quignon by M. Boucher de Perthes is really fossil. 2. That it was extracted by that gentleman himself from this virgin and undisturbed bed. 3d. That the flint implements which had been said to be fabricated by the workmen are incontestably ancient."

The specimen, which is said to resemble the jaw of the Esquimaux more than that of any other race now living, is thus described by Prof. Busk:

"The black coating was washed off readily by means of a sponge, and the residuary spots in the minute hollows were removed by the aid of a tooth-brush. The outer surface was tolerably smooth, presenting little indication of the erosion commonly seen in old buried bones. There was no appearance of dendritic patches either on the exterior or within, and no infiltration of metallic matter. The substance of the bone was dry and friable, especially towards the alveolar border, but, on the whole, it was tolerably firm under the saw, and the fresh section afforded a distinct odor of sawn bone. The internal cancellated structure was of a faint brownish tinge, and the cells were free from any incrustation. The most remarkable appearance observable in the section was the lining of the dental canal with a layer of fine gray sand, free from any admixture with the black metallic matrix which blocked up the orifice of the canal below the condyle. The section of the fang showed that the dentine, so far as exposed, was white, and in no respect different from that of a recent tooth. The enamel was white and brilliant. The socket towards the upper part was not completely filled by the fang, and the interval was partially occupied by black matrix and sandy particles."

